# Supplementary material for: Growth Potential of Listeria monocytogenes in Three Different Salmon Products
Source: Foods. 2020 Aug 3;9(8):1048. doi: 10.3390/foods9081048 (PMC7466188; doi:10.3390/foods9081048)
Supplement: Supplementary file 1 [file foods-09-01048-s001.pdf]

Figure S1. Cold growth curves

a

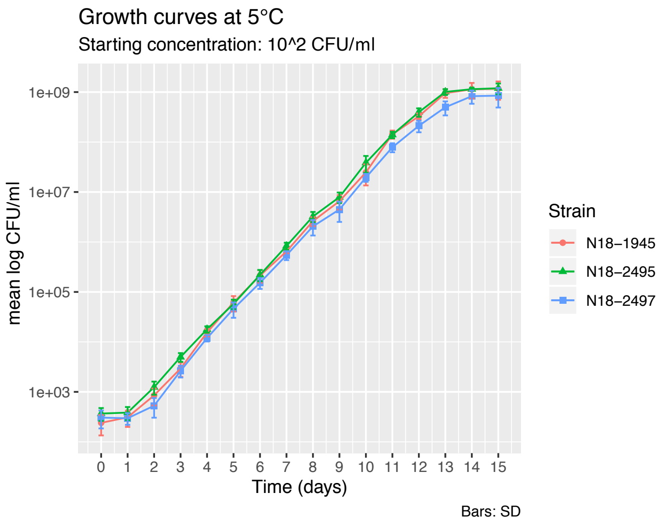

b

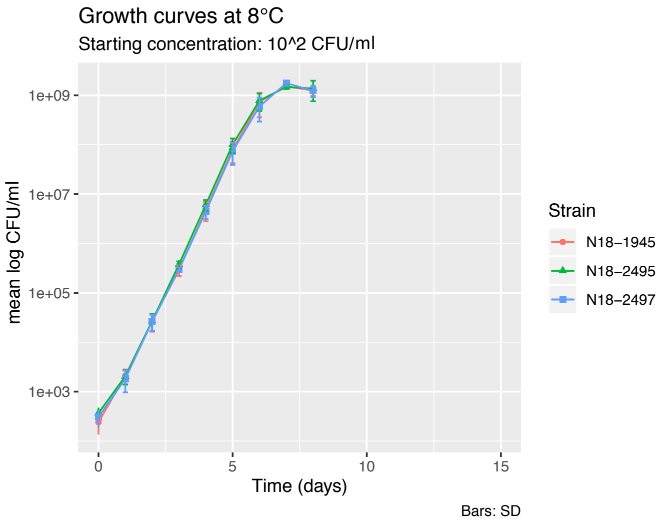

Cold-adapted early stationary phase cultures were prepared as described in the materials and methods section. These stationary phase cultures were used to inoculate 10 ml BHI with a target concentration of 100 CFU/ml, and growth was observed at (a) 5 °C and (b) 8 °C by direct plate counts on BHI agar. The curves represent three independent replicates.
